# Descending necrotising fasciitis of head and neck secondary to insect bite: report of a rare case

**DOI:** 10.11604/pamj.2021.38.271.28594

**Published:** 2021-03-16

**Authors:** Naman Kirit Pandya, Anendd Arroon Jadhav

**Affiliations:** 1 Department of Oral and Maxillofacial Surgery, Sharad Pawar Dental College and Hospital, Datta Meghe Institute of Medical Sciences, Sawangi, Maharastra, India

**Keywords:** Descending necrotising fasciitis, head, neck, infection, insect bite

## Image in medicine

A 30-year-old diabetic male reported with continuous fever, dyspnoea and rapidly progressive painful swelling over left side of face, ear, neck extending to involve skin of the chest following an unspecified insect bite over left ear since 2 days. The lesion extended to involve left face, ear, submental, bilateral submandibular region and descended progressively to involve cervicofacial and anterior chest wall with erythema at the advancing front (A, B). Further systemic evaluation revealed 320 mg/dl random blood sugar, 102.6° fever, HR 106 beats/min, blood pressure 74/50 mmHg with 22 respiratory cycles/min with 92% O_2_ saturation at atmospheric air. Further haematological evaluation demonstrated gross leukocytosis with presence of toxic granules and pencil cells in the peripheral smear indicative of sever sepsis/septic shock. The treatment was immediately instituted like supportive ventilation, insulin therapy, appropriate fluid, ionotropic support with empirical antibiotic therapy. Following initial optimization of the physical condition, the patient was scheduled for surgical debridement. However, the patient did not present with any clinical signs of improvement and within 6 hours after reporting, he succumbed to death following two episodes of cardiac arrest. Optimization of general condition of the patient, surgical debridement (i.e. fasciotomy and debridement) under appropriate empirical antibiotic coverage remains the critical tenet of management. The present report attempts to envisage the head and neck surgeon regarding unusual presentation with underlying comorbidities may rapidly transgress following a trivial injury to a life-threatening fatal event.

**Figure 1:**
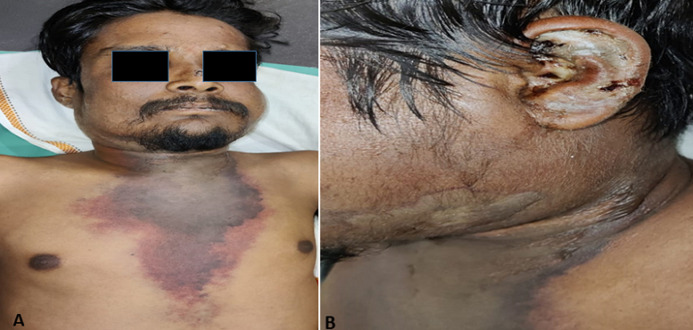
on presentation- A) a rapidly progressing dark brawny, dusky swelling along with necrosis of the skin descending down to involve cervicofacial and anterior chest wall with erythema at the advancing front; B) photograph of left ear on (B) presentation: site of insect bite with widespread edema and crusting of the per auricular skin with necrotic changes, descending downwards to facio-cervical region

